# Infarto Agudo do Miocárdio com Supradesnível de ST e Terapia de Reperfusão no Brasil: Dados do Registro ACCEPT

**DOI:** 10.36660/abc.20230863

**Published:** 2024-11-13

**Authors:** Luiz Eduardo Fonteles Ritt, Pedro Gabriel Melo de Barros e Silva, Eduardo Sahade Darzé, Renato Hideo Nakagawa Santos, Queila Borges de Oliveira, Otavio Berwanger, Luiz Alberto Piva e Mattos, Elizabete Silva dos Santos, Antonio Carlos Sobral Souza, Margaret Assad Cavalcante, Pedro Beraldo de Andrade, Fernando Carvalho Neuenschwander, Hugo Vargas, Jorge Ilha Guimarães, Jadelson Pinheiro de Andrade, Angelo Amato Vincenzo de Paola, Marcus Vinícius Bolívar Malachias, Dalton Bertolim Précoma, Fernando Bacal, Oscar Pereira Dutra

**Affiliations:** 1 Instituto D’Or de Ensino e Pesquisa Rio de Janeiro RJ Brasil Instituto D’Or de Ensino e Pesquisa, Rio de Janeiro, RJ – Brasil; 2 Hospital Cárdio Pulmonar Salvador BA Brasil Hospital Cárdio Pulmonar, Salvador, BA – Brasil; 3 Escola Bahiana de Medicina e Saúde Pública Salvador BA Brasil Escola Bahiana de Medicina e Saúde Pública, Salvador, BA – Brasil; 4 Hospital do Coração São Paulo SP Brasil Hcor, Hospital do Coração, São Paulo, SP – Brasil; 5 Brazilian Clinical Research Institute São Paulo SP Brasil Brazilian Clinical Research Institute, São Paulo, SP – Brasil; 6 Hospital Samaritano Paulista São Paulo SP Brasil Hospital Samaritano Paulista, São Paulo, SP – Brasil; 7 Centro Universitário São Camilo São Paulo SP Brasil Centro Universitário São Camilo, São Paulo, SP – Brasil; 8 Sociedade Brasileira de Cardiologia Rio de Janeiro RJ Brasil Sociedade Brasileira de Cardiologia, Rio de Janeiro, RJ – Brasil; 9 Instituto Dante Pazzanese de Cardiologia São Paulo SP Brasil Instituto Dante Pazzanese de Cardiologia, São Paulo, SP – Brasil; 10 Hospital São Lucas Aracaju SE Brasil Hospital São Lucas, Aracaju, SE – Brasil; 11 Universidade do Oeste Paulista Presidente Prudente SP Brasil Universidade do Oeste Paulista (Unoeste), Presidente Prudente, SP – Brasil; 12 Hospital Regional de Presidente Prudente Presidente Prudente SP Brasil Hospital Regional de Presidente Prudente, Presidente Prudente, SP – Brasil; 13 Faculdade de Medicina de Marília Marília SP Brasil Faculdade de Medicina de Marília, Marília, SP – Brasil; 14 Santa Casa de Marília Marília SP Brasil Santa Casa de Marília, Marília, SP – Brasil; 15 Hospital Vera Cruz Belo Horizonte MG Brasil Hospital Vera Cruz, Belo Horizonte, MG – Brasil; 16 Hospital São Vicente de Paulo Passo Fundo RS Brasil Hospital São Vicente de Paulo, Passo Fundo, RS – Brasil; 17 Hospital da Bahia Salvador BA Brasil Hospital da Bahia, Salvador, BA – Brasil; 18 Universidade Federal de São Paulo Escola Paulista de Medicina São Paulo SP Brasil Universidade Federal de São Paulo Escola Paulista de Medicina, São Paulo, SP – Brasil; 19 Faculdade de Ciências Médicas de Minas Gerais Belo Horizonte MG Brasil Faculdade de Ciências Médicas de Minas Gerais, Belo Horizonte, MG – Brasil; 20 Pontifícia Universidade Católica do Paraná Escola de Medicina Curitiba PR Brasil Pontifícia Universidade Católica do Paraná - Escola de Medicina, Curitiba, PR – Brasil; 21 Sociedade Hospitalar Angelina Caron – Cardiologia Campina Grande do Sul PR Brasil Sociedade Hospitalar Angelina Caron – Cardiologia, Campina Grande do Sul, PR – Brasil; 22 Universidade de São Paulo Faculdade de Medicina Hospital das Clínicas Instituto do Coração São Paulo SP Brasil Universidade de São Paulo Faculdade de Medicina Hospital das Clínicas Instituto do Coração, São Paulo, SP – Brasil; 23 Fundação Universitária de Cardiologia do Rio Grande do Sul Instituto de Cardiologia Porto Alegre RS Brasil Instituto de Cardiologia – Fundação Universitária de Cardiologia do Rio Grande do Sul, Porto Alegre, RS – Brasil

**Keywords:** Infarto do Miocárdio, Síndrome Coronariana Aguda, Registro Médico Coordenado

## Abstract

**Fundamento:**

Há carência de informações nacionais em relação a terapias utilizadas e evolução nos pacientes com síndrome coronária aguda com elevação de ST (SCACEST).

**Objetivos:**

Avaliar as terapias baseadas em evidência, a ocorrência de desfechos, uso de reperfusão e preditores para não receber reperfusão nos pacientes com SCACEST em um registro nacional multicêntrico.

**Métodos:**

Pacientes com SCACEST do Registro ACCEPT com até 12 horas de sintomas foram seguidos por 1 ano para ocorrência de eventos cardiovasculares maiores. Um p < 0,05 foi aplicado para todas análises.

**Resultados:**

Na análise de 1.553 pacientes, a taxa de reperfusão foi de 76,8%, variando de 47,5% na região Norte a até 80,5% na região Sudeste. A taxa de eventos cardiovasculares maiores foi de 12,5% em 1 ano. A prescrição de terapias baseadas em evidência na admissão hospitalar foi de 65,6%. A presença de hipertensão (odds ratio [OR] 1,47; intervalo de confiança [IC] 95% 1,11 a 1,96; p < 0,01), infarto agudo do miocárdio prévio (OR 1,81; IC 95% 1,32 a 2,48; p < 0,001) e as regiões Norte (OR 4,65; IC 95% 2,87 a 7,52; p < 0,001), Centro-Oeste (OR 4,02 IC 95% 1,26 a 12,7; p < 0,05) e Nordeste (OR 1,70; IC 95% 1,17 a 2,46; p < 0,01) foram preditores independentes de não utilização de terapia de reperfusão.

**Conclusões:**

No seguimento de 1 ano do Registro ACCEPT podemos verificar uma ampla variação dentre as regiões no que tange a aderência às melhores práticas de cuidado. Ser atendido nas regiões Norte, Centro-Oeste ou Nordeste, ter hipertensão arterial sistêmica ou infarto prévio foram preditores independentes de não utilização de terapia de reperfusão.

**Figure:**
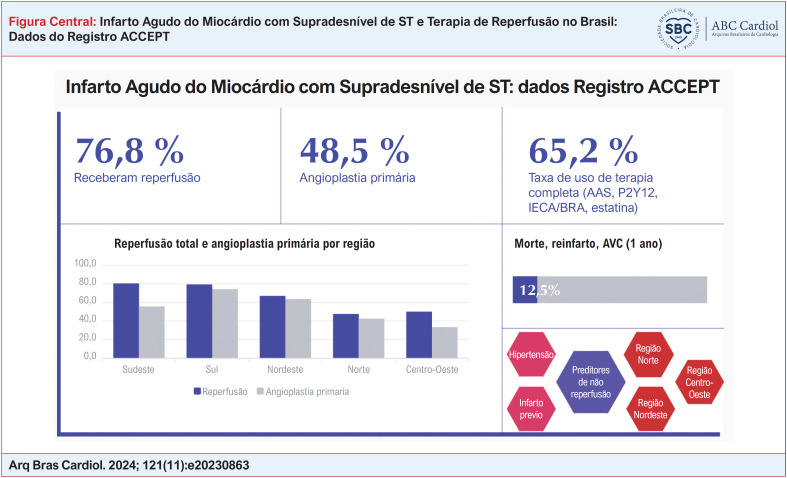


## Introdução

As doenças cardiovasculares representam a principal causa de mortalidade e incapacidade no Brasil e no mundo.^1-3^ A doença coronária na sua forma aguda, chamada síndrome coronária aguda (SCA), representa a causa isolada mais importante de óbito em dados epidemiológicos, mesmo em tempos de pandemia.^4,5^ Dentre o espectro de SCA, a síndrome coronária aguda com elevação de ST (SCACEST) é a que está relacionada a maiores taxas de complicação e mortalidade, sendo o melhor prognóstico diretamente dependente de um cuidado rápido e da implementação imediata de terapia de reperfusão.^6,7^ Entretanto, há falhas na aplicação das terapias baseadas em evidência em pacientes com SCA conforme identificado em registros prévios de prática clínica.^8-10^ O Brasil é um país continental e com disparidades no acesso à saúde, o que pode impactar diretamente no padrão de atendimento à SCA, especialmente em situações tempo-sensíveis como a SCACEST. O conhecimento do padrão de atendimento à SCACEST e os aspectos relacionados a um melhor prognóstico no âmbito nacional são informações necessárias para uma melhor organização dos serviços de saúde e para implementação de estratégias de melhoria no cuidado, seja no setor público ou no privado.

### Objetivos do estudo

Com base nos dados do Registro ACCEPT, um registro nacional conduzido pela Sociedade Brasileira de Cardiologia em 53 centros com representação em todas regiões do país, avaliamos o perfil e o prognóstico de pacientes atendidos por SCACEST no Brasil com os seguintes objetivos:

1. Descrever suas características clínicas, o perfil de tratamento e o prognóstico global e por região;
2. Avaliar a taxa de reperfusão global, por região e os tipos de reperfusão aplicados;
3. Avaliar os preditores de não utilização de terapia de reperfusão.

## Métodos

### Delineamento do estudo

O Registro ACCEPT (Acute Coronary Care Evaluation of Practice Registry) é um projeto idealizado pela Sociedade Brasileira de Cardiologia (SBC) cujos métodos foram previamente publicados.^10,11^ De forma resumida, trata-se de pesquisa prospectiva, voluntária, multicêntrica que reuniu 53 centros das 5 regiões brasileiras, assim distribuídos: Sudeste 50,9%, Nordeste 13,2%, Sul 24,5%, Centro-Oeste 5,7% e Norte 5,7%. O registro incluiu pacientes em centros hospitalares com assistência pública (Sistema Único de Saúde [SUS]), de saúde suplementar (operadoras de saúde) ou privados de agosto de 2010 até abril de 2014 assim distribuídos: SUS 55,8%, saúde suplementar 41,2% e privado 3%.

### Participantes do estudo

Foram incluídos pacientes na vigência do diagnóstico de SCA nas suas diferentes formas de apresentação: SCA sem elevação de ST (angina instável ou infarto agudo do miocárdio sem supradesnível do segmento ST) e também os casos de SCACEST. Foram excluídos pacientes transferidos de outras instituições com mais de 12 horas do início dos sintomas.

Na presente análise foram incluídos apenas pacientes com SCACEST, a qual foi definida pela presença de sintomas compatíveis com SCA por mais de 20 minutos associada a supradesnivelamento do segmento ST em 2 ou mais derivações contíguas sendo este > 2 mm nas derivações precordiais ou > 1 mm nas derivações periféricas ou novo bloqueio do ramo esquerdo com onda Q em 2 derivações contíguas. Pacientes fora da janela de reperfusão (com mais de 12 horas da apresentação) não foram incluídos na análise.

### Procedimentos do estudo e variáveis analisadas

Os procedimentos do estudo e variáveis analisadas no estudo ACCEPT foram previamente publicados.^10,11^

De forma sucinta, foram coletados dados demográficos, clínicos e dados do tratamento dos pacientes. Os pacientes foram seguidos em 7 dias ou até a alta hospitalar (o que ocorresse primeiro), 30 dias, 6 meses e 1 ano.

Tendo a característica de um estudo pragmático, a identificação de comorbidade dos pacientes (ex.: hipertensão arterial, dislipidemia) poderia ser realizada da seguinte forma: relato pelo paciente, uso de medicamento (anti-hipertensivo, hipolipemiante) ou avaliação do investigador (neste último, os centros foram orientados a seguirem as recomendações de critérios diagnósticos adotadas pelas diretrizes vigentes da Sociedade Brasileira de Cardiologia). Características do exame físico poderiam ser obtidas por mensuração direta (a obesidade foi definida por índice de massa corporal > 30 kg/m²). Demais critérios se basearam no registro em prontuário de uma variável coletada através de questionamento em entrevista (ex.: estresse, ex-tabagista se cessação > 6 meses). O esquema terapêutico baseado em evidência que foi considerado no ACCEPT não se modificou durante o estudo e se baseou em diretrizes vigentes. Este esquema terapêutico pode ser dividido da seguinte forma:

– Internação do evento índice: dupla antiagregação, anticoagulante parenteral, estatina e betabloqueador com adição da terapia de reperfusão nos casos de SCACEST.
– Terapia ambulatorial (pós-alta): dupla antiagregação, estatina, betabloqueador e inibidor da enzima conversora de angiotensina/bloqueador do receptor de angiotensina (IECA/BRA).

No que tange à terapia de reperfusão, os pacientes foram divididos entre 3 grupos: terapia de reperfusão mecânica (angioplastia primária), reperfusão farmacológica (trombolítico) e não reperfusão.

Os desfechos cardiovasculares de interesse analisados na população incluída foram: mortalidade cardiovascular, parada cardíaca não fatal, reinfarto e acidente vascular cerebral (AVC). Estes desfechos foram reportados pelo investigador de acordo com critérios recomendados sem utilização de um comitê independente para adjudicação de eventos.

### Análise estatística

Variáveis categóricas foram descritas como frequências absolutas ou relativas e comparadas pelo teste de qui-quadrado ou o teste exato de Fisher-Freeman-Halton.

Variáveis contínuas foram apresentadas como média e desvio padrão para as de distribuição normal ou mediana e intervalo interquartil para aquelas de distribuição não normal. A análise de histogramas foi aplicada para definição da distribuição. Médias entre dois grupos foram comparadas pelo teste de Student não pareado e entre mais de dois grupos pela análise de variância (ANOVA, *one-way analysis of variance*). A correção de Bonferroni foi utilizada para comparações múltiplas.

Modelos de regressão de Cox foram aplicados para determinar os preditores de não receber terapia de reperfusão. Análise de sobrevida de Kaplan-Meier foi aplicada para análise de sobrevida e para a comparação de curvas de sobrevida foi aplicada a estatística de log-rank.

As análises foram consideradas significantes se atingissem um p < 0,05. Todas as análises foram realizadas com auxílio do programa R versão 3.6.1.

## Resultados

Do total de 5.047 pacientes incluídos no registro, 1.714 (34%) tinham diagnóstico de SCACEST. Destes, 1.553 pacientes (31% do total) estavam dentro da janela de 12 horas de apresentação dos sintomas e formaram a população desta análise. Em um total de 147 pacientes (9,5%), não foi possível obter informação final de 12 meses.

### Características Basais

O perfil clínico-demográfico e de tratamento dos pacientes com SCACEST no geral e de acordo com a região, evidenciou uma maior prevalência do sexo masculino (73%), idade média de 60,7 ± 12,3 anos e um maior número de pacientes atendidos em serviços públicos (66%). Quanto a fatores de risco, dislipidemia estava presente em 43%, diabetes em 26% e infarto agudo do miocárdio prévio em 16%. Chama atenção um percentual maior de pacientes na região Nordeste com dislipidemia (59%) e com infarto agudo do miocárdio prévio (34%), além de uma média de idade maior nesta região (64 ± 12.9 anos), conforme a Tabela 1.

**Tabela 1:** Características demográficas, clínicas e tratamento para a população total e por região geográfica

		Total(1.553)	Sul(280)	Sudeste(1008)	Nordeste(173)	Norte(80)	Centro-Oeste(12)	p
**Idade (anos), média ± DP**		60,7 ± 12,3	58,7 ± 11	60,9 ± 12,5	64 ± 12,9	58,9 ± 10,9	62,4 ± 11,7	<0,001**
**Sexo masculino, n (%)**		1.129 (73%)	206 (73.6%)	726 (72%)	121 (70%)	67 (84%)	9 (75%)	0,179
**Dislipidemia, n (%)**		670 (43%)	112 (40%)	418 (41,5%)	102 (59%)	34 (42,5%)	4 (33,3%)	0,001
**IAM prévio, n (%)**		255 (16,4%)	39 (14%)	136 (13,5%)	60 (34,7%)	18 (22,5%)	2 (16,7%)	<0,001
**HAS, n (%)**		1.008 (65%)	173 (62%)	651 (64,6%)	120 (70%)	56 (70%)	8 (66,7%)	0,45
**Angina, n (%)**		378 (24,3%)	61 (22%)	211 (21%)	58 (33,5%)	44 (55%)	4 (33,3%)	<0,001
**AVC prévio, n (%)**		93 (6%)	20 (7,1%)	43 (4,3%)	15 (9%)	15 (19%)	0 (0%)	<0,001
**Diabetes, n (%)**		404 (26%)	68 (24,3%)	261 (26%)	47 (27,2%)	24 (30%)	4 (33,3%)	0,791
**ICC, n (%)**		81 (5,2%)	10 (3,6%)	27 (2,7%)	37 (21,4%)	7 (8,8%)	0 (0%)	<0,001
**ICP prévia, n (%)**		194 (12,5%)	43 (15,4%)	108 (10,7%)	31 (18%)	12 (15%)	0 (0%)	0,022
**Cirurgia de RM prévia, n (%)**		65 (4,2%)	11 (4%)	34 (3,4%)	13 (7,5%)	5 (6,2%)	2 (16,7%)	0,013
**Obesidade, n (%)**		459 (29,6%)	80 (28,6%)	271 (27%)	69 (40%)	36 (45%)	3 (25%)	<0,001
**Sedentarismo, n (%)**		877 (56,5%)	185 (66%)	519 (51,5%)	129 (75%)	40 (50%)	4 (33,3%)	<0,001
**Doença arterial periférica, n (%)**		120 (7,7%)	19 (6,8%)	61 (6,1%)	35 (20,2%)	5 (6,2%)	0 (0%)	<0,001
**Tabagismo, n (%)**								<0,001*
	Atual	605 (39%)	127 (45,4%)	406 (40%)	42 (24,3%)	28 (35%)	2 (16,7%)	
	Ex-tabagismo	346 (22%)	70 (25%)	203 (20%)	42 (24,3%)	28 (35%)	3 (25%)	
**Atendimento SUS, n (%)**		1.022 (66%)	257 (92%)	641 (64%)	33 (19,1%)	80 (100%)	11 (92%)	<0,001*

Valor de p: teste exato de Fisher.

* Teste qui-quadrado.

** Teste ANOVA.

AVC: acidente vascular cerebral; DP: desvio padrão; HAS: hipertensão arterial sistêmica; IAM: infarto agudo do miocárdio; n: número de indivíduos; ICC: insuficiência cardíaca congestiva; ICP: intervenção coronária percutânea; RM: revascularização do miocárdio; SUS: Sistema único de saúde.

### Terapia de reperfusão e outros medicamentos baseados em evidência

**Figura 1:**
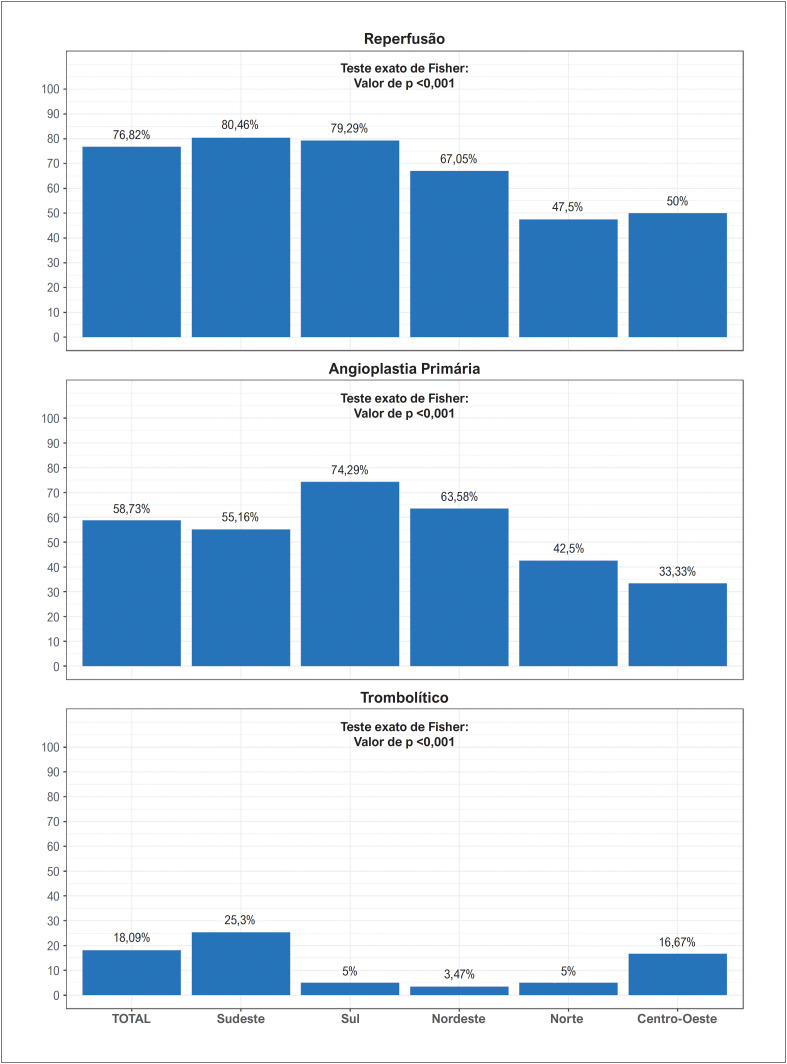
Percentual de reperfusão e tipos de reperfusão na população total e por região.

Os percentuais de reperfusão no geral e de acordo com a região geográfica estão dispostos na Figura 1. A taxa global de reperfusão foi de 76,8%, sendo mais prevalente o uso de angioplastia primária (58,7%). As regiões Sudeste e Sul apresentaram o maior índice de reperfusão (80,5% e 79,3%, respectivamente). As menores taxas de reperfusão foram observadas nas regiões Norte e Centro-Oeste (47,5% e 50%, respectivamente), conforme a Figura Central.

**Figura 2:**
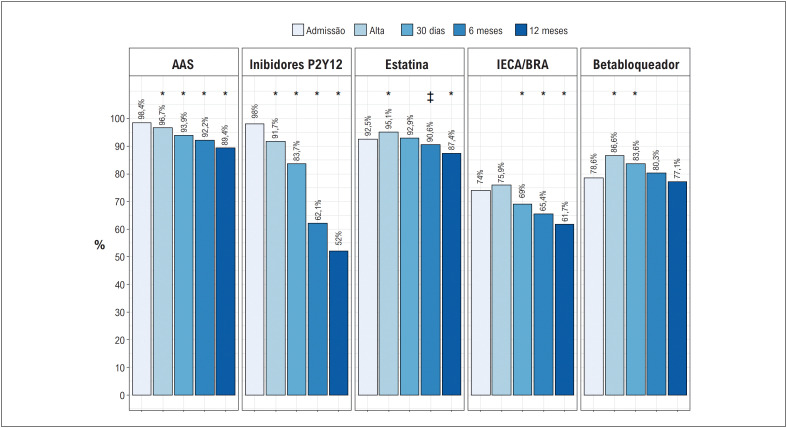
Adesão às terapias baseadas em evidência no seguimento de até 1 ano. AAS: ácido acetil salicílico; BRA: bloqueador do receptor de angiotensina; IECA: inibidor da enzima conversora de angiotensina.. *valor de p < 0,001 para comparação entre o seguimento e admissão. + valor de p < 0,05 para comparação entre o seguimento e admissão.

Na Tabela 2 estão dispostas as terapias utilizadas na admissão para a população total e por região e a Figura 2 traz o uso de terapias baseadas em evidência na admissão e no seguimento de até 1 ano. Terapia completa na admissão esteve presente em 65,6% dos casos em geral, tendo o menor nível na região Centro-Oeste. Houve uma redução ao longo do ano no uso de todas terapias, já se observando uma queda a partir do trigésimo dia, principalmente no uso de P2Y12, estatina e IECA/BRA.

**Tabela 2:** Utilização de medicamentos na fase de admissão dos pacientes com síndrome coronária aguda

		Total(1.553)	Sul(280)	Sudeste(1008)	Nordeste(173)	Norte(80)	Centro-Oeste(12)	p
**AAS, n (%)**		1.528 (98,4%)	275 (98,2%)	997 (99%)	170 (98,3%)	74 (92,5%)	12 (100%)	0,007
**Inibidor P2Y12, n (%)**		1.522 (98%)	273 (97,5%)	995 (98,7%)	169 (97,7%)	77 (96,2%)	8 (66,7%)	<0,001
	Clopidogrel	1.384 (89%)	242 (86,4%)	903 (90%)	156 (90%)	77 (96,2%)	6 (50%)	0,001
	Prasugrel	14 (1%)	0 (0%)	6 (0,6%)	6 (3,5%)	0 (0%)	2 (16,7%)	<0,001
	Ticagrelor	145 (9,3%)	35 (12,5%)	100 (9,9%)	9 (5,2%)	0 (0%)	1 (8,3%)	0,001
**Anticoagulante parenteral n (%)**		1.357 (87,4%)	221 (78,9%)	917 (91%)	134 (77,5%)	73 (91,2%)	12 (100%)	<0,001
	Enoxaparina	992 (64%)	147 (52,5%)	650 (64,5%)	112 (64,7%)	72 (90%)	11 (91,7%)	<0,001
	Fondaparinux	163 (10,5%)	0 (0%)	154 (15,3%)	9 (5,2%)	0 (0%)	0 (0%)	<0,001
	HNF	243 (15,6%)	80 (28,6%)	145 (14,4%)	16 (9,2%)	1 (1,2%)	1 (8,3%)	<0,001
**Inibidores da GP IIb/IIIa n (%)**		277 (17,8%)	62 (22%)	181 (18%)	26 (15%)	6 (7,5%)	2 (16,7%)	0,025
**Inibidor da ECA ou BRA n (%)**		1.149 (74%)	221 (78,9%)	775 (76,9%)	95 (54,9%)	51 (63,7%)	7 (58,3%)	<0,001
**Estatina n (%)**		1.436 (92,5%)	228 (81,4%)	960 (95,2%)	165 (95,4%)	73 (91,2%)	10 (83,3%)	<0,001
**Dupla terapia antiplaquetária (%)**		1.500 (96,6%)	268 (95,7%)	984 (97,6%)	166 (96%)	74 (92,5%)	8 (66,7%)	<0,001
**Terapia completa n (%)**		1.018 (65,6%)	144 (51,4%)	707 (70,1%)	109 (63%)	53 (66,2%)	5 (41,7%)	<0,001

Valor de p: Teste Exato de Fisher. Dupla terapia antiplaquetária: aspirina e inibidor de P2Y12. Terapia completa: dupla terapia antiplaquetária, anticoagulante parenteral, estatina e betabloqueador. AAS: ácido acetil salicílico; BRA: bloqueador do receptor de angiotensina ECA: enzima conversora de angiotensina; GP: glicoproteína; HNF: heparina não fracionada; n: número de indivíduos.

### Desfechos clínicos

**Figura 3:**
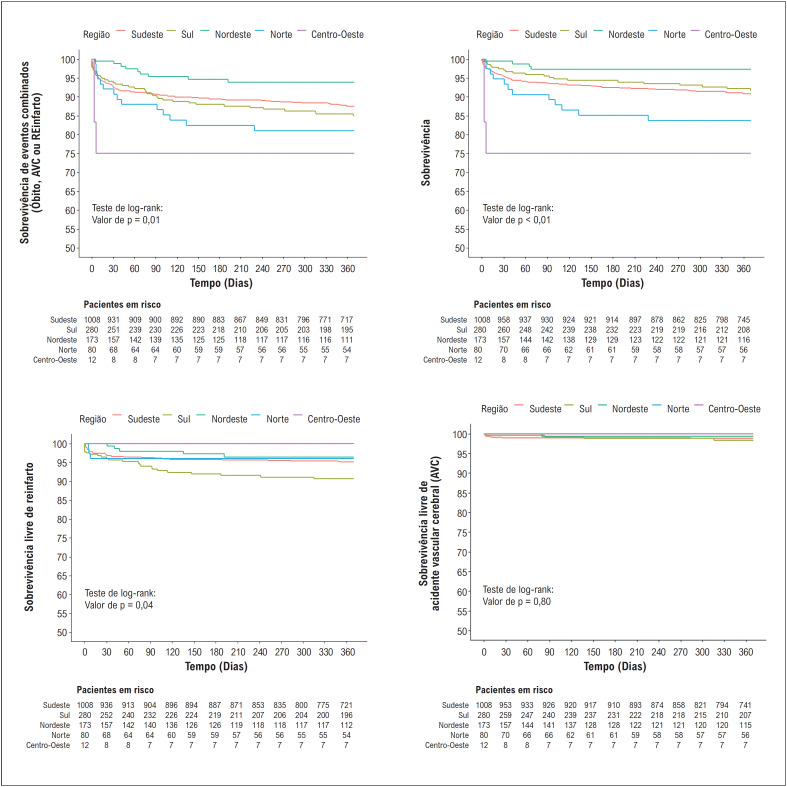
Curvas de sobrevida para os desfechos combinados (morte, reinfarto e AVC) e cada desfecho individual por região no seguimento de 1 ano.

A Figura 3 traz as curvas de sobrevida livre de eventos para os desfechos combinados (morte, reinfarto, AVC) e de forma individual na população total e em cada região no seguimento de 1 ano. A taxa global de eventos combinados foi de 12,5%, atingindo o menor índice na região Nordeste (5,2%) e os maiores índices nas regiões Norte e Centro-Oeste (17,5% e 25%, respectivamente), conforme a Tabela Suplementar 1 (Tabela S1).

### Terapia de reperfusão e preditores de não receber reperfusão

A Tabela Suplementar 2 (Tabela S2) traz a comparação dos grupos reperfusão e os que não receberam terapia de reperfusão. Na Tabela 3 o modelo final multivariado para os preditores de não receber terapia de reperfusão é apresentado. A presença de hipertensão (*odds ratio* [OR] 1,47; intervalo de confiança [IC] 95% 1,11 a 1,96; p < 0,01), infarto agudo do miocárdio prévio (OR 1,81; IC 95% 1,32 a 2,48; p < 0,001), as regiões Norte (OR 4,65; IC 95% 2,87 a 7,52; p < 0,001), Centro-Oeste (OR 4,02; IC 95% 1,26 a 12,7; p < 0,05) e Nordeste (OR 1,70; IC 95% 1,17 a 2,46; p < 0,01) foram preditores independentes de não receber terapia de reperfusão.

**Tabela 3:** Modelo final dos preditores independentes de não receber terapia de reperfusão

Coeficientes estimados do modelo de regressão logística para "Não receber terapia de reperfusão"						
Coeficiente	Coeficiente	Desvio padrão	Valor de p	OR	OR (IC 95%)	
					Inferior	Superior
Intercepto	-1,551	0,195	<0,001			
Região(Sul)	0,049	0,172	0,777	1,05	0,75	1,47
Região (Nordeste)	0,533	0,189	**0,005**	1,704	1,176	2,469
Região(Norte)	1,538	0,245	<**0,001**	4,653	2,879	7,521
Região (Centro-Oeste)	1,392	0,59	**0,018**	4,024	1,267	12,787
Sexo (Masculino)	-0,203	0,139	0,146	0,816	0,621	1,073
Dislipidemia (Sim)	-0,224	0,135	0,096	0,799	0,614	1,04
IAM prévio (Sim)	0,594	0,162	<**0,001**	1,812	1,32	2,487
HAS(Sim)	0,391	0,144	**0,007**	1,478	1,113	1,962
Sedentarismo (Sim)	0,199	0,13	0,126	1,22	0,945	1,575
Tabagismo (Atual)	-0,245	0,134	0,069	0,783	0,602	1,019

HAS: hipertensão arterial sistêmica; IAM: infarto agudo do miocárdio; IC: intervalo de confiança; OR: odds ratio.

## Discussão

O Registro ACCEPT é o maior registro nacional em SCA até o momento e, nesta análise, foi possível traçar as características, tratamento e prognóstico de pacientes com SCACEST elegíveis para terapia de reperfusão em uma população que contempla as 5 regiões do país. Foi possível verificarmos a taxa global de reperfusão e por região e o prognóstico no que tange a ocorrência de eventos cardiovasculares maiores (morte, reinfarto, AVE) no seguimento de até 1 ano, além de avaliar os preditores independentes para a não reperfusão. Estes dados poderão ser úteis para uma melhor definição de políticas de saúde para atingir um melhor cuidado ao paciente com SCACEST nas diversas regiões.^12-14^

### Taxas de reperfusão

Reperfusão foi realizada em 76,3%, mas variou de 47,5% na região Norte a 80,5% na Sudeste. Ao passo que as regiões Sul, Sudeste e Nordeste apresentaram taxas comparáveis a de registros norteamericanos^15^ e de países da Europa Ocidental,^16^ as taxas no Centro-Oeste e Norte são compatíveis com a de países do Leste Europeu e do Oriente Médio.^16^

O Brasil é um país continental com aspectos heterogêneos em termos de estrutura hospitalar e de logística de atendimento. Taxas de reperfusão estão diretamente associadas à criação de redes de atendimento ao paciente com SCA e à disponibilidade de serviços de atendimento terciário na localidade com capacidade para intervenção diuturnamente. Estes dados mostram uma necessidade iminente de uma atenção focada nessas regiões para entender os gargalos associados às baixas taxas de reperfusão que podem estar no campo não só da disponibilidade de serviços, mas também de recursos de realização e interpretação de ECG, diagnóstico clínico, disponibilidade de medicação trombolítica e de rotas de transferência para centros terciários além da logística de transporte. Experiências nacionais já foram publicadas com a implementação de redes de atendimento à SCACEST com resultados positivos, mostrando que ações integradas de implementação de rotinas e capacitação técnica são capazes de melhorar o cuidado nesta população.^17,18^

### Aderência à terapias baseadas em evidências

Outro parâmetro de nota no cuidado ao paciente com SCACEST é o uso de terapias que sejam classicamente relacionadas a um melhor desfecho no seguimento de médio e longo prazo dos pacientes. Foi possível observar que na admissão um percentual considerável dos pacientes estavam adequadamente tratados com uso de dupla-antiagregação, estatinas, betabloqueador e IECA/BRA, porém este percentual caiu gradualmente no seguimento de 30 dias e principalmente no seguimento de 6 meses, o que pode estar relacionado a uma menor ênfase na importância da manutenção destas medicações no seguimento ambulatorial a nível de atenção secundária ou mesmo em uma baixa aderência ou dificuldade na aquisição/acesso destas medicações. Protocolos e capacitações são mais prevalentes para o cuidado hospitalar. Dante do achado, acredita-se que ações para garantir que estas medicações sejam mantidas no seguimento ambulatorial são importantes para evitar eventos recorrentes.

### Prognóstico

A taxa global de eventos de 12,5% no seguimento de 1 ano, tendo sido maior na região Centro-Oeste com 25% de mortalidade em 1 ano. As taxas mais altas foram encontradas nas regiões Norte e Centro-Oeste, sendo estas as regiões com menores taxas de utilização de terapia de reperfusão e de uso de terapias baseadas em evidências; estes achados estão potencialmente relacionados ao maior número de desfechos e pior prognóstico nestas regiões. Em um estudo na cidade de Salvador-BA, uma taxa de eventos de 15% em 30 dias foi encontrada^18^ e, em um estudo no estado de Sergipe,^19^ os autores encontraram 12,8% em 30 dias. Dados europeus mostraram uma mortalidade intra-hospitalar que variava de 3% a 10%^16^ e o registro americano mostrou uma mortalidade intra-hospitalar de 7,9%.^15^

Chamou atenção a região Nordeste ter as menores taxas de eventos combinados (5,2%), apesar de ter uma taxa de utilização de reperfusão menor que as regiões Sul e Sudeste. Tal fato pode ter relação com outros fatores, como o tipo de centros envolvidos na região Nordeste, em que prevaleceram centros de atendimento de rede suplementar, e uma menor representatividade de centros de atenção pelo SUS com maior perda de seguimento nesta região em comparação com Sul e Sudeste (4% versus 30%).

### Preditores de não utilização de terapia de reperfusão

O tratamento da SCACEST tem na reperfusão imediata seu pilar fundamental e não deve ser retardada naqueles pacientes com apresentação na janela de 12 horas do início dos sintomas, seja com uso de terapia trombolítica ou por reperfusão mecânica via angioplastia primária. No Registro ACCEPT, a forma mais utilizada de reperfusão foi a angioplastia primária, o que pode estar relacionado à natureza dos centros participantes que de certa forma eram centros de referência local ou regional em atenção na cardiologia. O entendimento dos preditores de não utilização de terapia de reperfusão se faz importante como um guia nas ações e criação das redes de atenção ao infarto do miocárdio.

Foi possível verificar que as regiões Norte, Centro-Oeste e Nordeste estiveram relacionadas de forma independente à possibilidade de não receber terapia de reperfusão. Políticas específicas de criação de redes de atendimento ao paciente com SCA são prementes nestas regiões para que possamos estreitar o hiato e as diferenças em relação ao Sul e ao Sudeste. Além da questão geográfica, a história de infarto prévio e a hipertensão arterial sistêmica foram também relacionadas à chance de não receber terapia de reperfusão. O infarto prévio poderia estar relacionado a alterações no ECG que podem confundir e dificultar a interpretação como alterações de repolarização ou zonas inativas. Já a presença de hipertensão não controlada pode, por exemplo, levar a maior receio de iniciar terapia trombolítica em centros que não disponham de hemodinâmica 24/7 ou com equipes menos experientes no atendimento à SCA. Dados nos EUA,^15^ um país de dimensões continentais como o Brasil, também mostraram uma relação independente entre a região geográfica e a chance de não receber terapia de reperfusão. Infarto prévio e hipertensão também foram preditores independentes nos EUA.

### Pontos fortes e limitações

Um ponto forte do nosso estudo é o fato de abranger as 5 regiões do país e ser o estudo de registro com maior número de pacientes no país até a presente data e ainda o seguimento de 1 ano após o evento.

Nosso estudo tem limitações e ressaltamos que, apesar de termos representatividade em todas regiões, esta se concentrou na região Sudeste e a maioria dos centros estavam localizados nas capitais e em hospitais com capacidade de engajar-se em um registro de coleta de dados clínicos, o que de certa forma selecionou centros de melhor capacidade técnica, podendo assim a realidade ser diferente em centros menos experimentados. Como ressaltado, a perda de seguimento na região Nordeste pode de certa forma ter contribuído para um grau de subnotificação de eventos nessa região. O fato de não ter havido uma adjudicação de eventos pode ser, também, interpretado como uma limitação, porém o uso do desfecho como interpretado pelo próprio investigador traz uma noção mais próxima da rotina e da vida real.

O Registro ACCEPT finalizou seu período de coleta em 2014 e com seus dados tem-se a oportunidade de trazer informações importantes no cuidado do infarto agudo do miocárdio no Brasil, porém mesmo em um registro nacional e sob a égide da Sociedade Brasileira de Cardiologia algumas regiões como a Norte e a Centro-Oeste foram pouco representadas. Novos registros nacionais com uma abrangência maior não só de capitais, mas também de cidades no interior e em rincões mais distantes do país e em hospitais de atenção primária e secundária além dos terciários serão importantes para dados ainda mais reprodutivos. Os dados ora apresentados serão primordiais para estas comparações futuras. Com a evolução da coleta de dados direta de prontuários eletrônicos e com análise de big data essa tarefa pode ser de certa forma facilitada.

## Conclusão

No seguimento de 1 ano do Registro ACCEPT, foi possível determinar a taxa de reperfusão no infarto do miocárdio com supradesnível de ST, a aderência as principais medicações e o prognóstico. Foi verificado uma ampla variação dentre as regiões na aderência às melhores práticas. Pertencer a região Norte, Centro-Oeste ou Nordeste, ser hipertenso e ter infarto prévio foram preditores independentes de não reperfusão.

## *Material suplementar

Para informação adicional, por favor, clique aqui.
