# Supplementary material for: Infarto Agudo do Miocárdio com Supradesnível de ST e Terapia de Reperfusão no Brasil: Dados do Registro ACCEPT
Source: Arq Bras Cardiol. 2024 Nov 13;121(11):e20230863. [Article in Portuguese] doi: 10.36660/abc.20230863 (PMC11634312; doi:10.36660/abc.20230863)
Supplement: Supplementary file 1 [file 0066-782X-abc-121-11-e20230863-Suppl01.pdf]

Tabela S1 – Desfechos clínicos na população total e por região no seguimento de 1 ano

	**Total (1553)**	**Sul (280)**	**Sudeste (1008)**	**Nordeste (173)**	**Norte (80)**	**Centro-oeste (12)**	**p**
**Evento combinado (Morte, IAM, AVC) n (%)**	188 (12,1%)	39 (13,9%)	123 (12,2%)	9 (5,2%)	14 (17,5%)	3 (25%)	0,005
**Morte n (%)**	130 (8,3%)	21 (7,5%)	90 (8,9%)	4 (2,3%)	12 (15%)	3 (25%)	0,001
**IAM n (%)**	79 (5,1%)	24 (8,6%)	47 (4,6%)	5 (2,9%)	3 (3,75%)	0 (0%)	0,069
**AVC n (%)**	17 (1,1%)	4 (1,4%)	12 (1,2%)	1 (0,58%)	0 (0%)	0 (0%)	0,84

IAM, infarto agudo do miocárdio; AVC, acidente vascular cerebral. Valor de p: Teste exato de fisher.

Tabela S2 – Comparação dos pacientes submetidos ou não a algum tipo de terapia de reperfusão

**Variáveis**		**Terapia de Reperfusão**		
		**Não (n=360)**	**Sim (n=1193)**	**p**
**Atendimento**	SUS	240/360 (66,7%)	782/1193 (65,5%)	0,704
	Saúde Suplementar/Particular	120/360 (33,3%)	411/1193 (34,5%)	
**Região**	Sudeste	197/360 (54,7%)	811/1193 (68%)	<0,001
	Sul	58/360 (16,1%)	222/1193 (18,6%)	
	Nordeste	57/360 (15,8%)	116/1193 (9,7%)	
	Norte	42/360 (11,7%)	38/1193 (3,2%)	
	Centro-Oeste	6/360 (1,7%)	6/1193 (0,5%)	
**Idade (anos)**	Média ± dp	62 ± 12,7 (n=358)	60,4 ± 12,1 (n=1186)	**0,029**
**Sexo**	Masculino	253/360 (70,3%)	876/1193 (73,4%)	0,251
**História familiar de doença coronáriana**	Sim	146/360 (40,6%)	492/1193 (41,2%)	0,855
**Dislipidemia**	Sim	161/360 (44,7%)	509/1193 (42,7%)	0,505
**IAM prévio**	Sim	89/360 (24,7%)	166/1193 (13,9%)	**<0,001**
**HAS**	Sim	264/360 (73,3%)	744/1193 (62,4%)	**<0,001**
**Angina**	Sim	119/360 (33,1%)	259/1193 (21,7%)	**<0,001**
**AVC prévio**	Sim	28/360 (7,8%)	65/1193 (5,4%)	0,127
**Diabetes**	Sim	108/360 (30%)	296/1193 (24,8%)	0,055
**ICC**	Sim	30/360 (8,3%)	51/1193 (4,3%)	**0,004**
**Intervenção coronária percutânea**	Sim	61/360 (16,9%)	133/1193 (11,1%)	**0,005**
**Cirurgia de RM**	Sim	25/360 (6,9%)	40/1193 (3,4%)	**0,006**
**Obesidade Abdominal**	Sim	119/360 (33,1%)	340/1193 (28,5%)	**0,1**
**Sedentarismo**	Sim	220/360 (61,1%)	657/1193 (55,1%)	0,045
**Doença arterial periférica**	Sim	33/360 (9,2%)	87/1193 (7,3%)	0,26

**Tabagismo**	Nunca	147/360 (40,8%)	455/1193 (38,1%)	**0,005**
	Ex-tabagismo	97/360 (26,9%)	249/1193 (20,9%)	
	Atual	116/360 (32,2%)	489/1193 (41%)	
**Tratamento**	Tratamento Clínico	241/360 (66,9%)	NA	
	Angioplastia não primária	119/360 (33,1%)	NA	
	Só angioplastia primária	NA	912/1193 (76,4%)	
	Trombolítico	NA	114/1193 (9,6%)	
	Trombolítico e Angioplastia	NA	167/1193 (14%)	
**Trombolítico**	Sim	NA	281/1193 (23,6%)	
**Tempo agulha (minutos)**	mediana [quartis]		42 [17 - 105] (n=149)	
**Tipo de trombolítico**	SK	NA	100/281 (35,6%)	
	t-PA	NA	10/281 (3,6%)	
	TNK	NA	171/281 (60,9%)	
**Angioplastia**	Sim	119/360 (33,1%)	1079/1193 (90,4%)	<0,001
**Tempo porta-balão (minutos)**	mediana [quartis]	NA	93 [55,8 - 180] (n=952)	
**Tipo angioplastia**	Primária	NA	912/1079 (76,4%)	
**Stent**	Sim	102/119 (85,7%)	1032/1079 (95,6%)	<0,001
**Tipo stent**	Sem stent	17/119 (14,3%)	47/1079 (4,4%)	<0,001
	Stent farmacológico	14/119 (11,8%)	142/1079 (13,2%)	
	Stent não farmacológico	88/119 (73,9%)	890/1079 (82,5%)	
**Via de acesso**	Transradial	46/119 (38,7%)	431/1079 (39,9%)	0,844
**Valor de P: Teste Exato de Fisher.**				
*** Teste t-student não pareado**				
