# Supplementary material for: Infarto Agudo do Miocárdio com Supradesnível de ST e Terapia de Reperfusão no Brasil: Dados do Registro ACCEPT
Source: Arq Bras Cardiol. 2024 Nov 13;121(11):e20230863. [Article in Portuguese] doi: 10.36660/abc.20230863 (PMC11634312; doi:10.36660/abc.20230863)
Supplement: Translation

# Translation: Myocardial Infarction with ST Elevation and Reperfusion Therapy in Brazil: Data from the ACCEPT Registry

**DOI:** 10.36660/abc.20230863i

**Authors:** Luiz Eduardo Fonteles Ritt, Pedro Gabriel Melo de Barros e Silva, Eduardo Sahade Darzé, Renato Hideo Nakagawa Santos, Queila Borges de Oliveira, Otavio Berwanger, Luiz Alberto Piva e Mattos, Elizabete Silva dos Santos, Antonio Carlos Sobral Souza, Margaret Assad Cavalcante, Pedro Beraldo de Andrade, Fernando Carvalho Neuenschwander, Hugo Vargas, Jorge Ilha Guimarães, Jadelson Pinheiro de Andrade, Angelo Amato Vincenzo de Paola, Marcus Vinícius Bolívar Malachias, Dalton Bertolim Précoma, Fernando Bacal, Oscar Pereira Dutra

**Affiliations:** 1 Rio de Janeiro RJ Brazil Instituto D’Or de Ensino e Pesquisa, Rio de Janeiro, RJ – Brazil; 2 Salvador BA Brazil Hospital Cárdio Pulmonar, Salvador, BA – Brazil; 3 Salvador BA Brazil Escola Bahiana de Medicina e Saúde Pública, Salvador, BA – Brazil; 4 São Paulo SP Brazil Hcor, Hospital do Coração, São Paulo, SP – Brazil; 5 São Paulo SP Brazil Brazilian Clinical Research Institute, São Paulo, SP – Brazil; 6 São Paulo SP Brazil Hospital Samaritano Paulista, São Paulo, SP – Brazil; 7 São Paulo SP Brazil Centro Universitário São Camilo, São Paulo, SP – Brazil; 8 Rio de Janeiro RJ Brazil Sociedade Brasileira de Cardiologia, Rio de Janeiro, RJ – Brazil; 9 São Paulo SP Brazil Instituto Dante Pazzanese de Cardiologia, São Paulo, SP – Brazil; 10 Aracaju SE Brazil Hospital São Lucas, Aracaju, SE – Brazil; 11 Presidente Prudente SP Brazil Universidade do Oeste Paulista (Unoeste), Presidente Prudente, SP – Brazil; 12 Presidente Prudente SP Brazil Hospital Regional de Presidente Prudente, Presidente Prudente, SP – Brazil; 13 Marília SP Brazil Faculdade de Medicina de Marília, Marília, SP – Brazil; 14 Marília SP Brazil Santa Casa de Marília, Marília, SP – Brazil; 15 Belo Horizonte MG Brazil Hospital Vera Cruz, Belo Horizonte, MG – Brazil; 16 Passo Fundo RS Brazil Hospital São Vicente de Paulo, Passo Fundo, RS – Brazil; 17 Salvador BA Brazil Hospital da Bahia, Salvador, BA – Brazil; 18 São Paulo SP Brazil Universidade Federal de São Paulo Escola Paulista de Medicina, São Paulo, SP – Brazil; 19 Belo Horizonte MG Brazil Faculdade de Ciências Médicas de Minas Gerais, Belo Horizonte, MG – Brazil; 20 Curitiba PR Brazil Pontifícia Universidade Católica do Paraná - Escola de Medicina, Curitiba, PR – Brazil; 21 Campina Grande do Sul PR Brazil Sociedade Hospitalar Angelina Caron – Cardiologia, Campina Grande do Sul, PR – Brazil; 22 São Paulo SP Brazil Universidade de São Paulo Faculdade de Medicina Hospital das Clínicas Instituto do Coração, São Paulo, SP – Brazil; 23 Porto Alegre RS Brazil Instituto de Cardiologia – Fundação Universitária de Cardiologia do Rio Grande do Sul, Porto Alegre, RS – Brazil

**Keywords:** Myocardial Infarction, Acute Coronary Syndrome, Medical Record Linkage

## Abstract

**Background:**

There is a lack of information from Brazil regarding therapies used and outcomes in patients with acute coronary syndrome with ST elevation (STEMI).

**Objectives:**

To evaluate evidence-based therapies, occurrence of outcomes, reperfusion use, and predictors of not receiving reperfusion in patients with STEMI in a national multicenter registry.

**Methods:**

Patients with STEMI from the ACCEPT registry, with up to 12 hours of symptoms, were followed for 1 year for the occurrence of major adverse cardiovascular events. A significance level of p < 0.05 was applied for all analyses.

**Results:**

In the analysis of 1553 patients, the reperfusion rate was 76.8%, ranging from 47.5% in the North Region to 80.5% in the Southeast Region. The rate of major adverse cardiovascular events was 12.5% at 1 year. The prescription of evidence-based therapies at hospital admission was 65.6%. The presence of hypertension (odds ratio [OR] 1.47; 95% confidence interval [CI] 1.11 to 1.96; p < 0.01); prior acute myocardial infarction (OR 1.81; 95% CI 1.32 to 2.48; p < 0.001); and the North (OR 4.65; 95% CI 2.87 to 7.52; p < 0.001), Central-West (OR 4.02; 95% CI 1.26 to 12.7; p < 0.05), and Northeast Regions (OR 1.70; 95% CI 1.17 to 2.46; p < 0.01) were independent predictors of not receiving reperfusion therapy.

**Conclusion:**

In the 1-year follow-up of the ACCEPT Registry, we were able to verify a wide variation within Brazilian geographical regions regarding adherence to best care practices. The following were independent predictors of not receiving reperfusion therapy: being treated in the North, Central-West, and Northeast Regions; having systemic arterial hypertension; and prior infarction.

## Introduction

Cardiovascular diseases represent the leading cause of mortality and disability in Brazil and worldwide.^1-3^ Acute coronary heart disease, called acute coronary syndrome (ACS), represents the most important isolated cause of death in epidemiological data, even during the COVID-19 pandemic.^4,5^ Within the spectrum of ACS, acute coronary syndrome with ST elevation (STEMI) is associated with the highest rates of complications and mortality, and optimal prognosis is directly dependent on rapid care and immediate implementation of reperfusion therapy.^6,7^ However, there are failures in the application of evidence-based therapies in patients with ACS, as identified in previous registries of clinical practice.^8-10^ Brazil is a continent-sized country with disparities in access to health care, which can directly impact the standard of care for ACS, especially in time-sensitive situations such as STEMI. Knowledge about the standard of care for STEMI and aspects related to better prognosis in Brazil is necessary information for better organization of health services and for the implementation of strategies to improve care, in both the public or private sectors.

### Study objectives

Based on data from the ACCEPT Registry, a national registry conducted by the Brazilian Society of Cardiology in 53 centers representing all Brazilian regions, we evaluated the profile and prognosis of patients treated for STEMI in Brazil, with the following objectives:

1. To describe their overall and regional clinical characteristics, treatment profiles, and prognoses;
2. Evaluate the overall and regional reperfusion rate and types of reperfusion applied;
3. Evaluate predictors of not using reperfusion therapy.

## Methods

### Study design

The ACCEPT (Acute Coronary Care Evaluation of Practice Registry) is a project designed by the Brazilian Society of Cardiology whose methods have been previously published.^10,11^ In summary, it is a prospective, voluntary, multicenter study that brought together 53 centers from the 5 Brazilian regions, distributed as follows: Southeast 50.9%, Northeast 13.2%, South 24.5%, Central-West 5.7%, and North 5.7%. The registry included patients in hospital centers with public care (Brazilian Unified Health System [SUS]), supplementary health (health insurance companies), or private hospitals from August 2010 to April 2014, distributed as follows: SUS 55.8%, supplementary health 41.2%, and private 3%.

### Study participants

The registry included patients diagnosed with the following presentations of ACS: ACS without ST elevation (unstable angina or acute myocardial infarction without ST-segment elevation) as well as cases of STEMI. Patients transferred from other institutions more than 12 hours after onset of symptoms were excluded.

This analysis included only patients with STEMI, which was defined as the presence of symptoms compatible with ACS for more than 20 minutes associated with ST-segment elevation in 2 or more contiguous leads that were > 2 mm in the precordial leads or > 1 mm in the peripheral leads or new left bundle branch block with Q wave in 2 contiguous leads. Patients outside the reperfusion window (more than 12 hours after presentation) were not included in the analysis.

### Study procedures and variables analyzed

The study procedures and variables analyzed in the ACCEPT study have been previously published.^10,11^

In brief, patients’ demographic, clinical, and treatment data were collected. Patients were followed up at 7 days or until hospital discharge (whichever occurred first), 30 days, 6 months, and 1 year.

Given that it was a pragmatic study, patients’ comorbidities (e.g., hypertension, dyslipidemia) could be identified as follows: patient report, medication use (antihypertensive, lipid-lowering agents), or investigator assessment; in the latter case, centers were instructed to follow the diagnostic criteria recommendations adopted by the current Brazilian Society of Cardiology guidelines. Physical examination characteristics were obtained by direct measurement (obesity defined as body mass index > 30 kg/m^2^). Other criteria were based on medical records of a variable collected through questions in an interview (e.g., stress, ex-smoker if they had quit at least 6 months before). The evidence-based therapeutic regimen that was considered in the ACCEPT Registry was not modified during the study, and it was based on current guidelines. This therapeutic regimen can be divided as follows:

– Admission for index event: dual antiplatelet therapy, parenteral anticoagulant, statin, and beta blocker, with addition of reperfusion therapy in cases of STEMI
– Outpatient therapy (after discharge): dual antiplatelet therapy, statin, beta blocker, and angiotensin-converting enzyme inhibitor/angiotensin receptor blocker (ACEI/ARB).

With respect to reperfusion therapy, patients were divided into the following 3 groups: mechanical reperfusion therapy (primary angioplasty), pharmacological reperfusion (thrombolytic therapy), and no reperfusion.

The cardiovascular outcomes of interest analyzed in the population included were cardiovascular mortality, non-fatal cardiac arrest, reinfarction, and stroke. These outcomes were reported by the investigator according to recommended criteria, without the use of an independent event adjudication committee.

### Statistical analysis

Categorical variables were described as absolute or relative frequencies and compared using the chi-square test or the Fisher-Freeman-Halton exact test.

Continuous variables with normal distribution were presented as mean and standard deviation. Those with non-normal distribution were presented as median and interquartile range. Histogram analysis was applied to define the distribution. Means between two groups were compared using the unpaired Student's test, and those between more than two groups were compared using one-way analysis of variance (ANOVA). Bonferroni correction was used for multiple comparisons.

Cox regression models were applied to determine predictors of not receiving reperfusion therapy. Kaplan-Meier survival analysis was applied for survival analysis, and log-rank statistics were applied to compare survival curves.

Analyses were considered significant if they reached p < 0.05. All analyses were performed using the R program, version 3.6.1.

## Results

Of the total of 5047 patients included in the registry, 1714 (34%) were diagnosed with STEMI. Of these, 1553 patients (31% of the total) were within the 12-hour window of symptom onset, forming the population of this analysis. In a total of 147 patients (9.5%), it was not possible to obtain final 12-month information.

### Baseline characteristics

The clinical-demographic and treatment profile of patients with STEMI in general and according to region showed a higher prevalence of male sex (73%), with a mean age of 60.7 ± 12.3 years and a greater number of patients treated in public services (66%). Regarding risk factors, dyslipidemia was present in 43%, diabetes in 26%, and prior acute myocardial infarction in 16%. It is worth noting that there was a higher percentage of patients from the Northeast Region with dyslipidemia (59%) and prior acute myocardial infarction (34%), in addition to higher average age in the region (64 ± 12.9 years), as shown in Table 1.

**Table 1:** Demographic, clinical, and treatment characteristics for the total population and by geographic region

		Total(1553)	South(280)	Southeast (1008)	Northeast (173)	North](80)	Central-West (12)	p
**Age (years), mean ± SD**		60.7 ± 12.3	58.7 ± 11	60.9 ± 12.5	64 ± 12.9	58.9 ± 10.9	62.4 ± 11.7	<0.001**
**Male sex, n (%)**		1129 (73%)	206 (73.6%)	726 (72%)	121 (70%)	67 (84%)	9 (75%)	0.179
**Dyslipidemia, n (%)**		670 (43%)	112 (40%)	418 (41.5%)	102 (59%)	34 (42.5%)	4 (33.3%)	0.001
**Prior AMI, n (%)**		255 (16.4%)	39 (14%)	136 (13.5%)	60 (34.7%)	18 (22.5%)	2 (16.7%)	<0.001
**SAH, n (%)**		1.008 (65%)	173 (62%)	651 (64.6%)	120 (70%)	56 (70%)	8 (66.7%)	0.45
**Angina, n (%)**		378 (24.3%)	61 (22%)	211 (21%)	58 (33.5%)	44 (55%)	4 (33.3%)	<0.001
**Prior stroke, n (%)**		93 (6%)	20 (7.1%)	43 (4.3%)	15 (9%)	15 (19%)	0 (0%)	<0.001
**Diabetes, n (%)**		404 (26%)	68 (24.3%)	261 (26%)	47 (27.2%)	24 (30%)	4 (33.3%)	0.791
**CHF, n (%)**		81 (5.2%)	10 (3.6%)	27 (2.7%)	37 (21.4%)	7 (8.8%)	0 (0%)	<0.001
**Prior PCI, n (%)**		194 (12.5%)	43 (15.4%)	108 (10.7%)	31 (18%)	12 (15%)	0 (0%)	0.022
**Prior CABG, n (%)**		65 (4.2%)	11 (4%)	34 (3.4%)	13 (7.5%)	5 (6.2%)	2 (16.7%)	0.013
**Obesity, n (%)**		459 (29.6%)	80 (28.6%)	271 (27%)	69 (40%)	36 (45%)	3 (25%)	<0.001
**Sedentarism, n (%)**		877 (56.5%)	185 (66%)	519 (51.5%)	129 (75%)	40 (50%)	4 (33.3%)	<0.001
**Peripheral artery disease, n (%)**		120 (7.7%)	19 (6.8%)	61 (6.1%)	35 (20.2%)	5 (6.2%)	0 (0%)	<0.001
**Smoking, n (%)**								<0.001*
	Current	605 (39%)	127 (45.4%)	406 (40%)	42 (24.3%)	28 (35%)	2 (16.7%)	
	Ex-smoker	346 (22%)	70 (25%)	203 (20%)	42 (24.3%)	28 (35%)	3 (25%)	
**Treated through SUS, n (%)**		1.022 (66%)	257 (92%)	641 (64%)	33 (19.1%)	80 (100%)	11 (92%)	<0.001*

P value: Fisher's exact test.

* Chi-square test.

** ANOVA.

AMI: acute myocardial infarction; CABG: myocardial revascularization; CHF: congestive heart failure; n: number of individuals; PCI: percutaneous coronary intervention; SAH: systemic arterial hypertension; SD: standard deviation; SUS: Brazilian Unified Health System.

### Reperfusion therapy and other evidence-based medications

The percentages of reperfusion in general and according to geographic region are shown in Figure 1. The overall reperfusion rate was 76.8%, and use of primary angioplasty (58.7%) was most prevalent. The Southeast and South Regions had the highest reperfusion rates (80.5% and 79.3%, respectively). The lowest reperfusion rates were observed in the North and Central-West Regions (47.5% and 50%, respectively), as shown in the Central Figure.

**Figure 1:** Percentage of reperfusion and types of reperfusion in the total population and by region.

Table 2 displays the therapies used at admission for the total population and by region, and Figure 2 displays the use of evidence-based therapies at admission and in the follow-up period of up to 1 year. Complete therapy at admission was present in 65.6% of cases in general, with the lowest level in the Central-West Region. There was a reduction throughout the year in the use of all therapies, with a decrease already observed after the thirtieth day, mainly in the use of P2Y12 inhibitor, statin, and ACEI/ARB.

**Table 2:** Medication use during the admission phase in patients with acute coronary syndrome

		Total (1553)	South (280)	Southeast (1008)	Northeast (173)	North (80)	Central-West (12)	p
**ASA, n (%)**		1528 (98.4%)	275 (98.2%)	997 (99%)	170 (98.3%)	74 (92.5%)	12 (100%)	0.007
**P2Y12 inhibitor, n (%)**		1522 (98%)	273 (97.5%)	995 (98.7%)	169 (97.7%)	77 (96.2%)	8 (66.7%)	<0.001
	Clopidogrel	1384 (89%)	242 (86.4%)	903 (90%)	156 (90%)	77 (96.2%)	6 (50%)	0.001
	Prasugrel	14 (1%)	0 (0%)	6 (0.6%)	6 (3.5%)	0 (0%)	2 (16.7%)	<0.001
	Ticagrelor	145 (9.3%)	35 (12.5%)	100 (9.9%)	9 (5.2%)	0 (0%)	1 (8.3%)	0.001
**Parenteral anticoagulant, n (%)**		1357 (87.4%)	221 (78.9%)	917 (91%)	134 (77.5%)	73 (91.2%)	12 (100%)	<0.001
	Enoxaparin	992 (64%)	147 (52.5%)	650 (64.5%)	112 (64.7%)	72 (90%)	11 (91.7%)	<0.001
	Fondaparinux	163 (10.5%)	0 (0%)	154 (15.3%)	9 (5.2%)	0 (0%)	0 (0%)	<0.001
	UFH	243 (15.6%)	80 (28.6%)	145 (14.4%)	16 (9.2%)	1 (1.2%)	1 (8.3%)	<0.001
**GP IIb/IIIa inhibitors, n (%)**		277 (17.8%)	62 (22%)	181 (18%)	26 (15%)	6 (7.5%)	2 (16.7%)	0.025
**ACEI or ARB, n (%)**		1149 (74%)	221 (78.9%)	775 (76.9%)	95 (54.9%)	51 (63.7%)	7 (58.3%)	<0.001
**Statin, n (%)**		1436 (92.5%)	228 (81.4%)	960 (95.2%)	165 (95.4%)	73 (91.2%)	10 (83.3%)	<0.001
**Dual antiplatelet therapy, n (%)**		1500 (96.6%)	268 (95.7%)	984 (97.6%)	166 (96%)	74 (92.5%)	8 (66.7%)	<0.001
**Complete therapy, n (%)**		1018 (65.6%)	144 (51.4%)	707 (70.1%)	109 (63%)	53 (66.2%)	5 (41.7%)	<0.001

P value: Fisher's exact test. Dual antiplatelet therapy: aspirin and P2Y12 inhibitor. Complete therapy: dual antiplatelet therapy, parenteral anticoagulant, statin, and beta blocker. ACEI: angiotensin-converting enzyme inhibitor; ARB: angiotensin receptor blocker; ASA: acetylsalicylic acid; GP: glycoprotein; n: number of individuals; UFH: unfractionated heparin.

**Figure 2:** Adherence to evidence-based therapies in up to 1 year of follow-up. ACEI: angiotensin-converting enzyme inhibitor; ARB: angiotensin receptor blocker; ASA: acetylsalicylic acid. * P value < 0.001 for comparison between follow-up and admission. + P value < 0.05 for comparison between follow-up and admission.

### Clinical outcomes

Figure 3 displays the event-free survival curves for the combined outcomes (death, reinfarction, stroke) and individually in the total population and in each region over the 1-year follow-up. The overall combined event rate was 12.5%, reaching the lowest rate in the Northeast Region (5.2%) and the highest rates in the North and Central-West Regions (17.5% and 25%, respectively), as shown in Supplementary Table 1 (Table S1).

**Figure 3:** Survival curves for combined outcomes (death, reinfarction, and stroke) and each individual outcome by region at 1-year follow-up.

### Reperfusion therapy and predictors of not receiving reperfusion

Supplementary Table 2 (Table S2) compares the reperfusion groups and those that did not receive reperfusion therapy. Table 3 displays the final multivariate model for predictors of not receiving reperfusion therapy. The presence of hypertension (odds ratio [OR] 1.47; 95% confidence interval [CI] 1.11 to 1.96; p < 0.01); prior acute myocardial infarction (OR 1.81; 95% CI 1.32 to 2.48; p < 0.001); and the North (OR 4.65; 95% CI 2.87 to 7.52; p < 0.001), Central-West (OR 4.02; 95% CI 1.26 to 12.7; p < 0.05), and Northeast (OR 1.70; 95% CI 1.17 to 2.46; p < 0.01) Regions were independent predictors of not receiving reperfusion therapy.

**Table 3:** Final model of independent predictors of not receiving reperfusion therapy

Estimated coefficients of the logistic regression model for "Not receiving reperfusion therapy"						
Coefficient	Coefficient	Standard deviation	p value	OR	OR (95%CI)	
					Lower	Upper
Intercept	-1.551	0.195	<0.001			
Region(South)	0.049	0.172	0.777	1.05	0.75	1.47
Region (Northeast)	0.533	0.189	**0.005**	1.704	1.176	2.469
Region(North)	1.538	0.245	<**0.001**	4.653	2.879	7.521
Region (Central-West)	1.392	0.59	**0.018**	4.024	1.267	12.787
Sex(Male)	-0.203	0.139	0.146	0.816	0.621	1.073
Dyslipidemia (Yes)	-0.224	0.135	0.096	0.799	0.614	1.04
Prior AMI (Yes)	0.594	0.162	<**0.001**	1.812	1.32	2.487
SAH(Yes)	0.391	0.144	**0.007**	1.478	1.113	1.962
Sedentarism (Yes)	0.199	0.13	0.126	1.22	0.945	1.575
Smoking (Current)	-0.245	0.134	0.069	0.783	0.602	1.019

AMI: acute myocardial infarction; CI: confidence interval; OR: odds ratio; SAH: systemic arterial hypertension.

## Discussion

The ACCEPT Registry is the largest Brazilian ACS registry to date. In this analysis, it was possible to outline the characteristics, treatment, and prognosis of patients with STEMI who were eligible for reperfusion therapy in a population that includes all 5 regions of Brazil. It was also possible to verify the overall and regional reperfusion rate and prognosis regarding the occurrence of major adverse cardiovascular events (death, reinfarction, stroke) in up to 1 year of follow-up, in addition to evaluating the independent predictors of not receiving reperfusion. These data can be useful for better defining health policies to achieve better care for patients with STEMI in diverse regions.^12-14^

### Reperfusion rates

Reperfusion was performed in 76.3% of cases, but it ranged from 47.5% in the North to 80.5% in the Southeast. While the South, Southeast, and Northeast Regions presented rates comparable to those of registries from North America^15^ and Western Europe,^16^ the rates in the Central-West and North were compatible with those from countries in Eastern Europe and the Middle East.^16^

Brazil is a continent-sized country with heterogeneous aspects in terms of hospital structure and care logistics. Reperfusion rates are directly associated with the creation of care networks for patients with ACS and the availability of tertiary care services in the location with the capacity for intervention around the clock. These data show an imminent need for focused attention in these regions to understand the bottlenecks associated with low reperfusion rates that may not only be in the field of service availability, but also of resources for performing and interpreting ECGs, clinical diagnosis, availability of thrombolytic medication, and transfer routes to tertiary centers, in addition to transportation logistics. Brazilian experiences have already been published with the implementation of care networks for STEMI with positive results, showing that integrated actions for implementing routines and technical training are capable of improving care for this population.^17,18^

### Adherence to evidence-based therapies

Another noteworthy parameter in the care of patients with STEMI was the use of therapies that are classically related to better outcomes in the medium- and long-term follow-up of patients. We observed that, upon admission, a considerable percentage of patients were adequately treated with the use of dual antiplatelet therapy, statin, beta blocker, and ACEI/ARB; however, this percentage gradually decreased in the 30-day follow-up and especially in the 6-month follow-up, which may be related to lesser emphasis on the importance of maintaining these medications in outpatient follow-up at the secondary care level or even low adherence or difficulties in acquiring/accessing these medications. Protocols and training are more prevalent for hospital care. Given this finding, we believe in the importance of actions to ensure that these medications are maintained during outpatient follow-up in order to avoid recurrent events.

### Prognosis

The overall event rate was 12.5% in the 1-year follow-up. It was higher in the Central-West region, with 25% mortality in 1 year. The highest rates were found in the North and Central-West Regions, which had the lowest rates of reperfusion therapy and use of evidence-based therapies. These findings are potentially related to the greater number of outcomes and worse prognosis in these regions. A study in the city of Salvador, Bahia found an event rate of 15% in 30 days,^18^ and a study in the Brazilian state of Sergipe^19^ found a rate of 12.8% in 30 days. European data have shown in-hospital mortality rates ranging from 3% to 10%,^16^ and the United States registry showed an in-hospital mortality rate of 7.9%.^15^

It was noteworthy that the Northeast Region had the lowest rates of combined events (5.2%), even though it had a lower reperfusion rate than the South and Southeast Regions. This fact may be related to other factors, such as the type of centers involved in the Northeast Region, where supplementary network care centers prevailed, and a lower representation of SUS centers, with greater loss to follow-up in this region compared to the South and Southeast (4% versus 30%).

### Predictors of not using reperfusion therapy

Immediate reperfusion is a fundamental pillar in the treatment of STEMI, and it should not be delayed in patients presenting within the 12-hour window of symptom onset, whether with the use of thrombolytic therapy or mechanical reperfusion via primary angioplasty. In the ACCEPT Registry, the most commonly used form of reperfusion was primary angioplasty, which may be related to the nature of the participating centers, which were, to a certain extent, local or regional reference centers for cardiology care. It is important to understand the predictors of not using reperfusion therapy as a guide for actions and the creation of myocardial infarction care networks.

It was possible to verify that the North, Central-West, and Northeast Regions were independently related to the possibility of not receiving reperfusion therapy. Specific policies for the creation of networks to care for patients with ACS are urgently needed in these regions, in order to narrow the gap and differences in relation to the South and Southeast. In addition to the geographic issue, history of prior infarction and systemic arterial hypertension were also related to the chances of not receiving reperfusion therapy. Previous infarction could be related to ECG changes that can confound and complicate the interpretation as repolarization changes or inactive zones. On the other hand, the presence of uncontrolled hypertension may, for example, lead to a greater concern in initiating thrombolytic therapy in centers that do not have 24/7 hemodynamics or with teams that are less experienced in ACS care. Data from the United States,^15^ a continent-sized country like Brazil, also showed an independent relationship between geographic region and the chances of not receiving reperfusion therapy. Prior infarction and hypertension were also independent predictors in the United States.

### Strengths and limitations

Our study's strengths include the facts that it covers the 5 regions of Brazil and that it is the registry study with the largest number of patients in the country to date, in addition to the 1-year follow-up after the event.

Our study has some limitations. We underscore that, although we have representation from all regions, it was concentrated in the Southeast Region, and the majority of the centers were located in capital cities and hospitals with the capacity to engage in a registry with clinical data collection, which to a certain extent selected centers with better technical capacity; the reality may thus be different in less experienced centers. As highlighted, the loss of follow-up in the Northeast Region may have contributed to a certain degree of underreporting of events in the region. The fact that there was no adjudication of events can also be interpreted as a limitation. However, the use of the outcome as interpreted by the investigators offers a notion that is closer to routine and real life.

The ACCEPT Registry finalized its collection period in 2014, and its data provided an opportunity to access important information regarding acute myocardial infarction care in Brazil. However, even in a national registry supported by the Brazilian Society of Cardiology, some regions such as the North and Central-West were underrepresented. New national registries with a broader scope, encompassing not only capital cities, but also locations in the interior and more distant corners of the country including primary and secondary care hospitals, in addition to tertiary care, will be important for even more productive data. The data presented here will be essential for these future comparisons. With the development of direct data collection from electronic medical records and big data analysis, this task can be facilitated to a certain extent.

## Conclusion

In the 1-year follow-up of the ACCEPT Registry, it was possible to determine the reperfusion rate in STEMI, adherence to the main medications, and prognosis. A wide variation was observed between regions regarding adherence to best practices. The following were independent predictors of non-reperfusion: belonging to the North, Central-West or Northeast Regions; having hypertension; and previous infarction.

## *Supplemental Materials

For additional information, please click here.
